# RhBMP-2 loaded 3D-printed mesoporous silica/calcium phosphate cement porous scaffolds with enhanced vascularization and osteogenesis properties

**DOI:** 10.1038/srep41331

**Published:** 2017-01-27

**Authors:** Cuidi Li, Chuan Jiang, Yuan Deng, Tao Li, Ning Li, Mingzheng Peng, Jinwu Wang

**Affiliations:** 1 School of Biomedical Engineering, Shanghai Jiao Tong University, Shanghai, China; 2 Shanghai Key Laboratory of Orthopaedic Implants, Shanghai Ninth People’s Hospital Affiliated Shanghai Jiao Tong University School of Medicine, Shanghai, China; 3 Department of Orthopaedics, Sun Yat-sen Memorial Hospital, Sun Yat-sen University, 107 West Yanjiang Road, Guangzhou, China

## Abstract

A major limitation in the development of effective scaffolds for bone regeneration has been the limited vascularization of the regenerating tissue. Here, we propose the development of a novel calcium phosphate cement (CPC)-based scaffold combining the properties of mesoporous silica (MS) with recombinant human bone morphogenic protein-2 (rhBMP-2) to facilitate vascularization and osteogenesis. Specifically, the development of a custom MS/CPC paste allowed the three-dimensional (3D) printing of scaffolds with a defined macroporous structure and optimized silicon (Si) ions release profile to promote the ingrowth of vascular tissue at an early stage after implantation in support of tissue viability and osteogenesis. In addition, the scaffold microstructure allowed the prolonged release of rhBMP-2, which in turn significantly stimulated the osteogenesis of human bone marrow stromal cells *in vitro* and of bone regeneration *in vivo* as shown in a rabbit femur defect repair model. Thus, the combination MS/CPC/rhBMP-2 scaffolds might provide a solution to issues of tissue necrosis during the regeneration process and therefore might be able to be readily developed into a useful tool for bone repair in the clinic.

Over the last several decades, researchers have developed different kinds of bone repair scaffolds, utilizing cells and growth factors to enhance the osteoinduction properties for better healing effect^1^,^2^. However, poor angiogenesis within tissue-engineered implants has been regarded as a primary challenge limiting the clinical introduction of bone tissue-engineering implants. Localized necrosis and implant failure have been found to occur because of lack of inner blood vessels to facilitate the transport of nutrition and removal of waste^3^,^4^. Thus, greater attention has been paid towards the design and fabrication of bone tissue engineering implants with internal vessel networks. In particular, vessel networks with branching structures including major vessel conduits to small capillary beds inside the scaffold have been found to play an important role in promoting both osteogenesis and angiogenesis^5^,^6^.

Recently, bioactive mesoporous calcium silicate/calcium phosphate cement (MCS/CPC) scaffolds with well interconnected macropores have been successfully fabricated by our laboratory using three-dimensional (3D) extrusion-based printing^7^. Owing to the inherent self-setting property of calcium phosphate cement, MCS/CPC scaffolds could be printed at room temperature without sintering afterward and exhibited higher mechanical strength property than other 3D printed inorganic scaffolds. Furthermore, the introduction of mesoporous silica (MS)-based particles improved the biodegradation and effectively prolonged the printable period of the printing paste.

Further more, the micro-structure of MS would be expected to promote Si ions release to the surrounding media. Si is considered as an essential element for healthy bone and vascular development^8^,^9^. Released Si ions from MS powders have been verified to stimulate the osteogenic differentiation of human bone marrow stromal cells (hBMSCs)^10^ and enhance the proangiogenesis of endothelial cells (ECs)^11^,^12^,^13^. A Si ions concentration between 0.6 and 2 μg/mL has been found to stimulate human aortic endothelial cell proliferation and the gene expression of KDR, bFGFR1, and TGFbR3^14^. Accordingly, we hypothesized that the highly interconnected macropores and released Si ions might benefit vascular ingrowth inside the scaffolds. The transport of nutrients to the implant site facilitated by abundant newly formed blood vessels might further promote the osteogenesis process. However, the formation mode of new blood vessels inside the straight pores fabricated using the 3D printing technique with the concomitant release of Si ions has not yet been verified and analysed.

Currently, the process of bone regeneration with biomaterials still requires long periods of time. RhBMP-2 is used extensively in clinical applications for the efficient stimulation of bone formation^15^. The combination of scaffolds and rhBMP-2 has been shown to achieve more effective bone regeneration *in vivo* in comparison with that obtained using biomaterial matrices alone^16^,^17^. An ideal carrier for rhBMP-2 should be designed to achieve the controllable release of the growth factor at proper rate with good bioactivity and stability. Among potential carriers, mesoporous silica with an ordered mesopore structure is widely used as carrier for drugs and proteins because of its high specific surface area^18^,^19^,^20^. Therefore, the function of this carrier after being doped in the printed scaffolds deserves to be examined in detail.

Considering these component points, the efficacy of the combination of MS, interconnected pore structure, and osteoinductive growth factors in guiding vascularization and osteogenesis is worthy of further investigation. In this study, we fabricated MS/CPC scaffolds with highly interconnected macropores and bioactivated them using rhBMP-2. The effects of MS and rhBMP-2 on the osteogenic differentiation of hBMSCs and the vascularization of human umbilical vein endothelial cells (HUVECs) were investigated *in vitro*. Furthermore, a femur defect rabbit model was applied for transplanting the MS/CPC and MS/CPC/rhBMP-2 scaffolds, using CPC scaffolds as a control. The *in vivo* vascularization and osteogenesis processes effected by the different scaffolds were subsequently evaluated in considerable detail.

## Results

### Physical properties of the printing pastes

**Figure 1:**
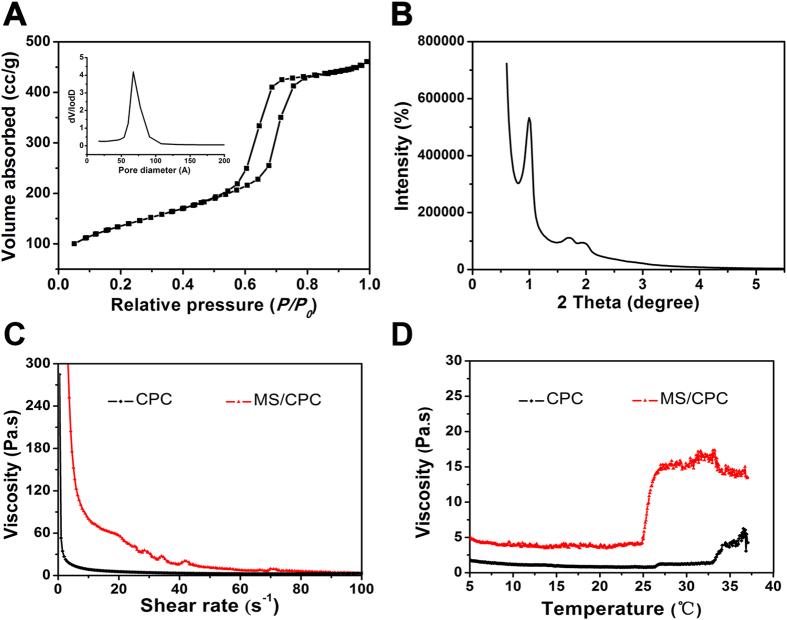
Effect of doped MS on the physical properties of the printing pastes. (**A**) N_2_ adsorption–desorption isotherms and pore size distribution of MS. (**B**) Small-angle X-ray diffraction of MS. (**C**) Viscosities of CPC and MS/CPC paste under different loading conditions. (**D**) Viscosities of CPC and MS/CPC paste under different temperatures at a fixed shear rate.

The N_2_ adsorption-desorption isotherms of the MS powder are presented in Fig. 1A. The curve of MS powder can be identified as a type IV isotherm with a H1 hysteresis loop, which is typical for mesoporous materials with a narrow pore size distribution centred at 6.8 nm. Small-angle X-ray diffraction (SAXRD) was applied to monitor the mesoporous structure of the MS powder. As illustrated in Fig. 1B, distinct diffraction peaks indexed to 100 reflections and weak signal peaks indexed to 110 reflections could be clearly identified, indicating the ordered mesoscopic symmetry of MS. It was observed from Fig. 1C that the viscosity of both CPC and MS/CPC decreased with the increase of shear rate and exhibited pseudoplastic flow behaviour. Thus, it was indicated the shear-thinning characteristic of CPC paste would not be changed upon addition of MS. Figure 1D shows the viscosity curves of CPC and MS/CPC pastes. It can be seen that with the addition of MS, the MS/CPC pastes demonstrated much higher viscosity than the CPC pastes while the temperature increased from 4 °C to 37 °C. The viscosity of the MS/CPC pastes dramatically increased during 25 °C to 27 °C and maintained around 15 Pas. The viscosity of CPC slightly increased to approximately 4 Pas when the temperature reached 34 °C.

### Microstructure and morphology of the MS/CPC scaffolds

**Figure 2:**
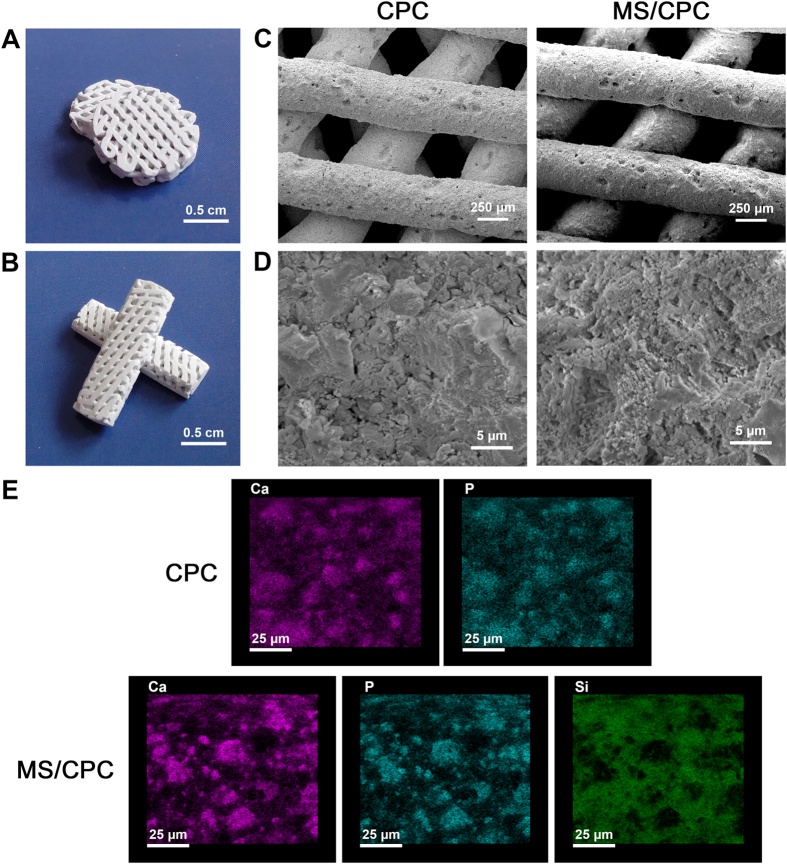
Microstructure and morphology of the MS/CPC scaffolds. (**A**,**B**) Digital camera photographs of MS/CPC scaffolds for *in vitro* (**A**) and *in vivo* (**B**) experiments. Scanning electron micrographs of CPC and MS/CPC (**C**) ×150, (**D**) ×10 K. (**E**) Element distribution on the surface of CPC and MS/CPC scaffolds.

Figure 2A,B show the digital photographs of the MS/CPC scaffolds used for the *in vitro* (Fig. 2A) and *in vivo* (Fig. 2B) experiments. It can be observed that both CPC and MS/CPC scaffolds obtained uniform and interconnected pore structures as intended (Fig. 2C). The topology structure and element distribution on the surface of the scaffolds was detected using SEM (Fig. 2D) and EDS (Fig. 2E) analysis. The results showed that the MS/CPC scaffolds had a more rough surface than CPC scaffolds, which may be attributable to the difference of particle size between MS and CPC powders. EDS analysis indicated that Si atoms were uniformly dispersed on the surface of the scaffolds as were Ca and P atoms, which verified that MS powders with much smaller particle size dispersed evenly among larger CPC powders.

### Cell viability of HUVECs and the capillary tube formation assay

**Figure 3:**
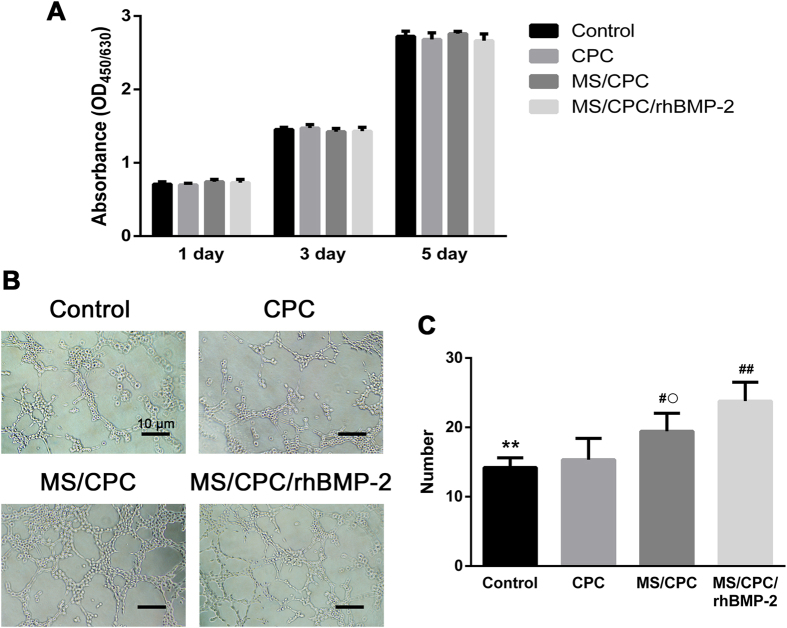
*In vitro* vascularization evaluations. (**A**) Effects of different extractions on the proliferation of HUVECs after 1, 3, and 5 d of culture. (**B**) Microscopy images of tube formation of HUVECs after culturing with different extraction media on Matrigel for 5 h at 37 °C. (**C**) Number of tubes formed per field with different extractions. **P < 0.01, control vs. MS/CPC and MS/CPC/rhBMP-2; ^#^*P* < 0.05, ^##^*P* < 0.01 CPC vs. MS/CPC and MS/CPC/rhBMP-2; ^○^*P* < 0.05 MS/CPC vs. MS/CPC/rhBMP-2.

Figure 3A shows the viability of HUVECs in different extraction media over the entire culture period. The results showed that the cells exhibited good growth and survival in the extraction medium of each group and that the incorporation of rhBMP-2 showed no significant effect on the cell viability. Next, the capillary tube formation capability of HUVECs cultured with different extractions was investigated by microscopy (Fig. 3B) and quantified in terms of number of capillary tubes per field by a blinded observer (Fig. 3C). HUVECs cultured with standard culture medium (the control group) and the CPC scaffold extraction proliferated and formed into a capillary-like network with few tubes after 5 h on Matrigel. Compared to the control and CPC groups, cells cultured in extraction media of MS/CPC and MS/CPC/rhBMP-2 scaffolds both formed networks with significantly increased numbers of tubes per field (*P* < 0.01). Additionally, cells cultured in the MS/CPC/rhBMP-2 extraction medium demonstrated better capillary tube formation ability than those cultured in MS/CPC extraction (*P* < 0.05).

### Viability and attachment of hBMSCs

**Figure 4:**
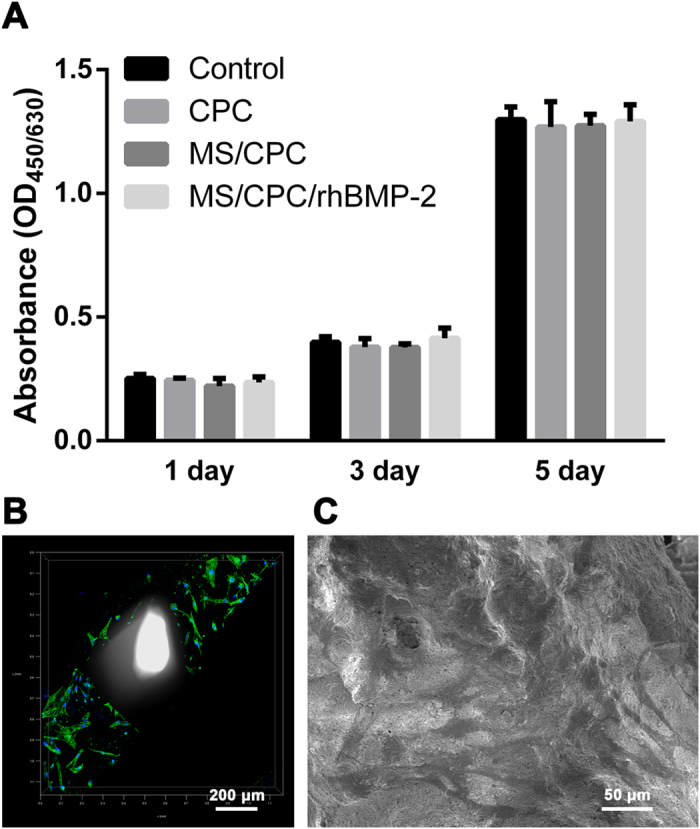
Effects of MS on cell biocompatibility. (**A**) Proliferation of hBMSCs in the extraction medium of MS/CPC scaffolds during culture for 1, 3, and 5 d assessed with the CCK-8 assay. (**B**) Confocal laser scanning microscopy images of the cytoskeleton stained with FITC-Phalloidin (green) and nuclei stained with DAPI (blue) of hBMSC attachment and morphology on the MS/CPC scaffolds after 3 d of culture. White columns inside the scaffold were caused by the light penetrated the macropores from confocal laser scanning microscope.(**C**) SEM images of hBMSCs attached on the MS/CPC scaffolds after 3 d of culture.

Figure 4A shows that the viability of hBMSCs was high over the entire culture period. No significant difference was observed among the CPC, MS/CPC, and MS/CPC/rhBMP-2 scaffolds. The attachment and morphology of hBMSCs on the surface of the MS/CPC scaffolds were observed by confocal microscopy (Fig. 4B) and SEM (Fig. 4C). It can be seen that hBMSCs spread well and attached closely onto the surface of the MS/CPC scaffolds. Cells with well-defined microfilaments and an intact nucleus were seen to have grown around and into the macropores.

### Osteogenic differentiation of hBMSCs

**Figure 5:**
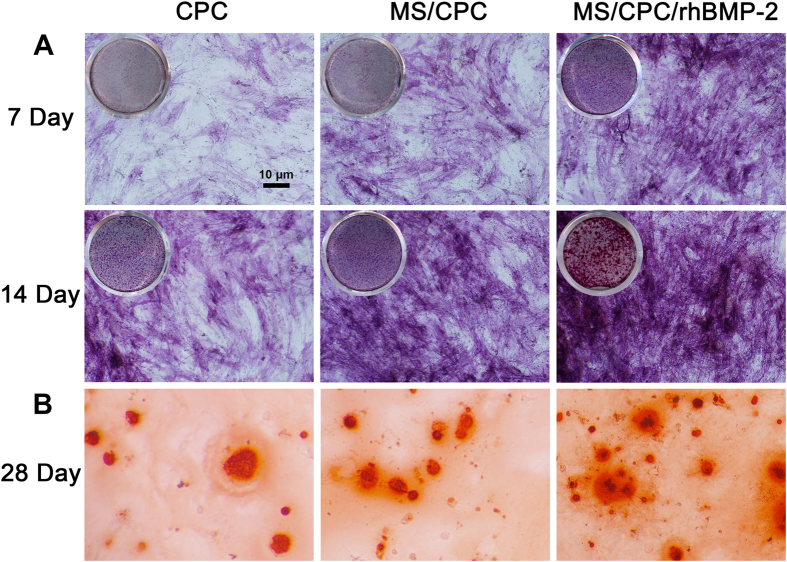
Effects of Si ions and rhBMP-2 release on the osteogenic differentiation of hBMSCs. (**A**) ALP staining of hBMSCs after 7 and 14 days of culture in extraction medium of CPC, MS/CPC, and MS/CPC/rhBMP-2. (**B**) Alizarin Red-S staining of hBMSCs mineralization after 28 d of culture in extraction medium of CPC, MS/CPC or MS/CPC/rhBMP-2 scaffolds.

The ALP activity and calcium deposition capability of hBMSCs were monitored to understand the influence of the culture media containing released components from the scaffolds on the osteogenic differentiation behaviour of the cells. As shown in Fig. 5, the ALP-positive area of the MS/CPC/rhBMP-2 scaffold group was apparently larger than that of the other two groups after culture of 7 and 14 days, whereas the MS/CPC scaffolds group showed more positive area than the CPC scaffold group. The results from Alizarin Red S staining showed a similar trend: the corresponding areas of bone nodule formation of the MS/CPC/rhBMP-2 scaffolds were largest among all the groups and the CPC group showed the least bone nodule formation.

### Vessel formation of scaffolds in rabbit femoral defects

**Figure 6:**
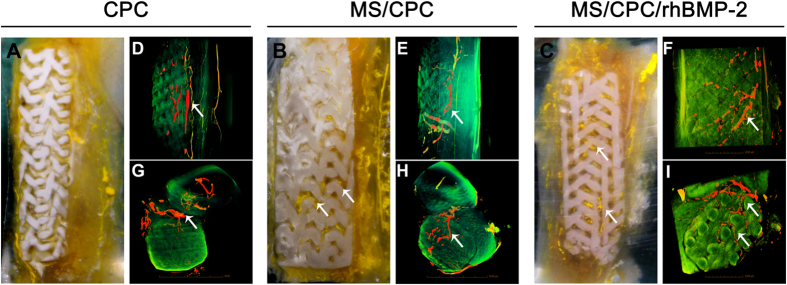
Effects of MS and rhBMP-2 released on vascularization. The (**A**–**C**) Digital camera photographs of PMMA-embedded blocks from longitudinal sections and 3D reconstructed μCT images of blood vessels from (**D**–**F**) side view and (**G**–**I**) top view of CPC, MS/CPC, and MS/CPC/rhBMP-2 scaffolds after 4 weeks of implantation. White arrow: newly formed blood vessels.

The effects of scaffolds on vascularization were studied using microangiography and the micro-computerised tomography (μCT) assay (Fig. 6). From the digital photographs shown in Fig. 6A–C of samples embedded in polymethylmethacrylate (PMMA) after 4 weeks of implantation, abundant vessels labelled by yellow-coloured Microfil were vividly displayed inside the macropores of the MS/CPC and MS/CPC/rhBMP-2 scaffolds and around the defects whereas few blood vessels were seen inside the CPC scaffolds. The 3D reconstructed images of new blood vessels (red and yellow) formed in the macropores and around the scaffolds (green) are also presented in Fig. 6D–I. The same trend was observed as in the digital photographs. Furthermore, from the top view images, the newly formed vessels could be seen to have grown straight along the pore structure and deep inside the middle of the MS/CPC and MS/CPC/rhBMP-2 scaffolds and, from the side view images, to have grown through the entire MS-doped scaffolds from top to bottom.

### Bone regeneration of scaffolds in rabbit femoral defects

**Figure 7:**
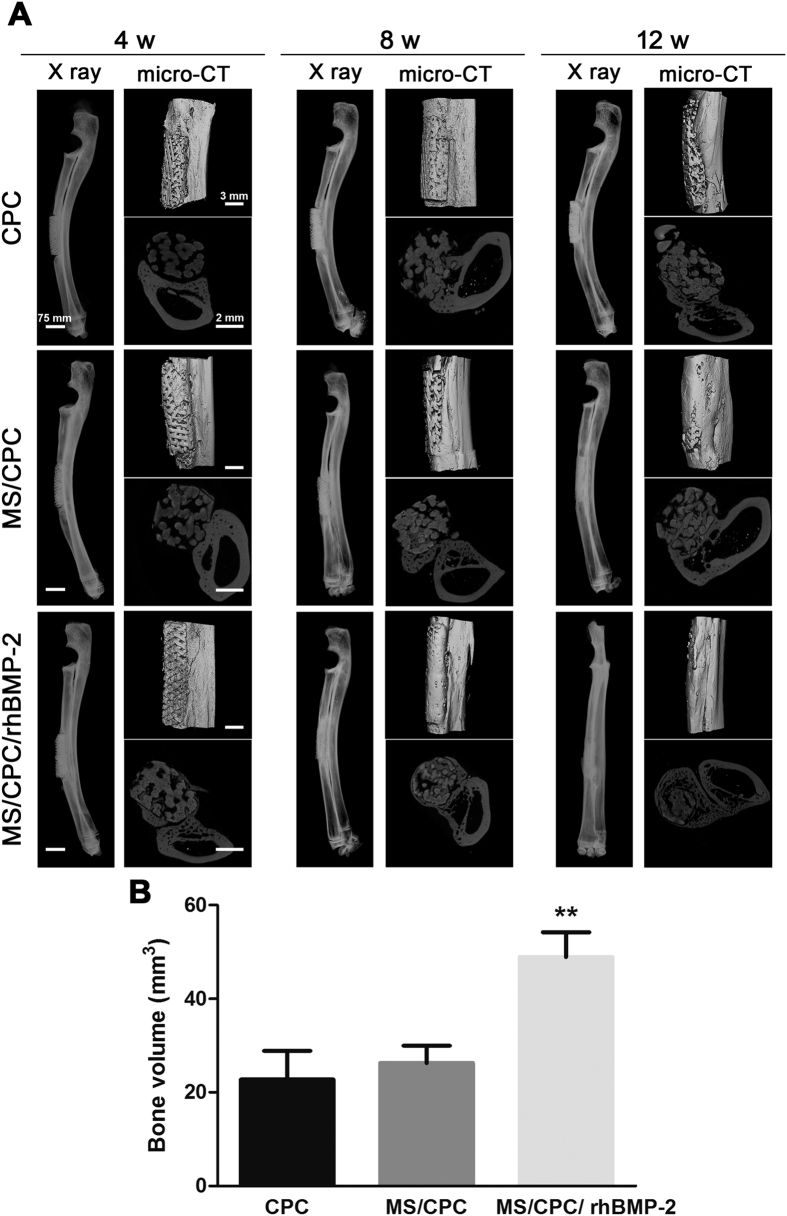
Effects of MS and rhBMP-2 on osteogenesis. (**A**) X ray images, 3D reconstructed μCT images from longitudinal sections (parallel to the long axis of bone), and slices from cross section (vertical to the long axis of bone) of CPC, MS/CPC, and MS/CPC/rhBMP-2 scaffolds at 4, 8, and 12 weeks post-implantation. (**B**) Newly formed bone volume at the defect site implanted with CPC, MS/CPC, and MS/CPC/rhBMP-2 scaffolds **P < 0.01, MS/CPC/rhBMP-2 vs. CPC and MS/CPC scaffolds.

X-ray, μCT, and reconstructed 3D images of bone regeneration at the defect sites are presented in Fig. 7A. The best circumferential cortical regeneration was found in the MS/CPC/rhBMP-2 scaffold group. It could be observed that the newly formed bone gradually grew around and inside the scaffolds, functionally rebridging the defect after 12 weeks of implantation. The repair effect of MS/CPC scaffolds was slightly better than that of CPC scaffolds with respect to repair rate and integrity whereas the CPC scaffolds displayed the slowest healing rate. The newly formed bone volumes in the defects after 12 weeks of implantation were calculated and are listed in Fig. 7B. It was shown that the MS/CPC/rhBMP-2 scaffolds contained the highest bone volume (*P* < 0.01), which supported our observation from the images.

### Histological analysis

**Figure 8:**
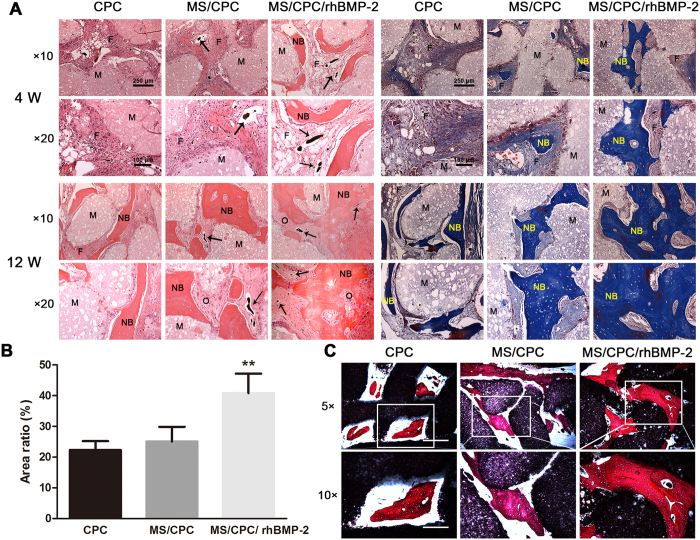
Histological evaluation of longitudinal sections of orthotopic bone formation within CPC, MS/CPC and MS/CPC/rhBMP-2 scaffolds. (**A**) HE and Masson trichrome staining (x10, x20) after 4 and 12 weeks of implantation. M: materials, NB: newly formed bones, F: fibrous tissue, O: osteoid, Black arrow: newly formed blood vessels. (**B**) Quantitative analysis of the new bone area in HE stained sections after 12 weeks of implantation. **P < 0.01, MS/CPC/rhBMP-2 vs. CPC and MS/CPC scaffolds. (**C**) VG staining (x5:bar = 500 μm, x10:bar = 250 μm) after 12 weeks of implantation.

Histological analysis was performed to provide a more detailed analysis of the tissue formation process. The HE and Masson staining results of bone defects sites implanted with scaffolds for 4 and 12 weeks are shown in Fig. 8A. After implantation for 4 weeks, osteogenesis was barely detected in the CPC and MS/CPC scaffolds except for some fibrous connective tissue. During the regeneration process, newly formed bone tissue grew gradually into the central region as well as into most macropores of the scaffolds and bound tightly with the material. After implantation for 12 weeks, the newly formed bone was quantified; the results are shown in Fig. 8B. It was revealed that a greater quantity of bone regenerated upon MS/CPC/rhBMP-2 scaffolds implantation than from the other two groups (*P* < 0.01). Newly formed blood vessels could be clearly observed inside the macropores of MS/CPC and MS/CPC/rhBMP-2 scaffolds because of the Microfil we injected. Sections histologically stained using Van Gieson’s stain after implantation for 12 weeks are presented in Fig. 8C. Newly formed mature bone tissue could be seen to have formed inside the macropores of all three groups. However, in comparison to the other groups, MS/CPC/rhBMP-2 scaffolds displayed the best osteogenesis, with new bone occupying the macropores.

## Discussion

Despite substantial advances, vascularization of large bone grafts remains a major challenge that has held back the clinical translation of engineered bone constructs^21^. Bone tissue engineering implants able to provide both accelerated osteogenesis and vascularization are regarded as an important solution. The present study developed rhBMP-2 loaded MS/CPC scaffolds with a hierarchical pore structure. The synergistic effects of pore structure, Si ions, and growth factor on osteogenesis and vascularization was systematically studied.

First, a novel MS/CPC printing paste was developed. As there exists a relatively large difference between particle sizes of the MS and CPC powders, the even dispersion of MS powder between CPC powder led to a more dense and viscous MS/CPC printing paste. Thus, the generated MS/CPC paste was able to maintain flexibility and continuity under the tension during printing, and the lower parts of the scaffolds on the platform were less able to collapse under the pressure of the upper parts. With these CPC and MS/CPC pastes, we successfully bioprinted scaffolds with interconnected macropores of approximately 300 μm, a size that had been suggested to enhance cell ingrowth, migration, and nutrient transport into the inner part of the scaffolds and hence to promote tissue ingrowth^22^,^23^.

The MS doped scaffolds provided a microstructure from which Si ions were gradually released following immersion in an aqueous solution. Previously, it has been shown that Si ions at appropriate concentrations could stimulate the osteogenic differentiation of BMSCs and angiogenesis of ECs via increased gene expression of proangiogenic cytokine receptors and up-regulated downstream signalling events^13^,^24^,^25^,^26^. Consistent with this finding, our results showed that the extraction medium of MS/CPC scaffolds exerted a positive influence on the osteogenic differentiation of hBMSCs and markedly promoted tube formation of HUVECs *in vitro*. These results were similar to those previously reported and verified that the content of MS doped into the scaffolds was appropriate.

Vascularization at an early stage after implantation is known to provide the necessary oxygen and nutrients for cell and new tissue ingrowth and for accelerated bone reconstruction^27^,^28^,^29^,^30^. Importantly, it has been illustrated that vascularization comprises two parts: one represents perfusable blood vessels for the restoration of blood flow to the site of injury and the other consists of a microvascular system for providing blood throughout the entire scaffold, which supports osteogenesis and osseointegration^21^. First of all, our *in vivo* studies indicated that MS/CPC scaffolds played a positive role towards promoting vascularization at an early stage after bone trauma. Additionally, the formation of new blood vessels in the implantation site analysed via the Microfil experiment was interesting. The digital photographs indicated that greater numbers of newly formed blood vessels could be seen in the MS/CPC group than in the CPC group. From the μCT results after 4-week implantation, it was observed that abundant vasculature formed around pure CPC scaffolds but that little could be observed inside the scaffolds; thus, only the first part of vascularization was represented. Notably, newly formed vasculature not only grew around the MS/CPC and MS/CPC/rhBMP-2 scaffolds but also along the interconnected macropores and throughout the entire implant, demonstrating that both parts of vascularization were reconstructed. The mechanism underlying the difference in the neovascularization processes was not studied in depth here; however, this will be the subject of further research. Silicate biomaterials, for example, have been reported to enhance vascularization through stimulating the expression of VEGF, an important angiogenic growth factor^31^,^32^. We hypothesized that the generation of VEGF consequent to our protocol then triggered the migration of endothelial cells, with invasion of their tip cells into the inner part of the scaffolds shortly after implantation and subsequent proliferation of stalk cells, which thereby stimulated the formation of vascular networks^33^,^34^. RhBMP-2 has also been reported to indirectly promote angiogenesis by stimulating the endogenous expression of VEGF^35^,^36^,^37^. However, the *in vivo* results from our study showed that the MS/CPC/rhBMP-2 group exhibited only a slightly greater number of newly formed blood vessels than the MS/CPC group, which suggested that Si ions might play a primary role in neovascularization.

Evidence has been presented that BMP-2 promotes bone formation via several different mechanisms such as osteoblast differentiation, chemoattraction, angiogenesis, and cell signalling at the initiation of fracture healing. A primary concern in developing our protocol was to prolong the releasing rate and osteogenesis function of the growth factor via the microstructure of the MS/CPC scaffolds. Our results demonstrated that the MS/CPC scaffolds acted as suitable carriers for rhBMP-2. The MS/CPC/rhBMP-2 scaffolds showed a slower release speed of the protein than CPC scaffolds (Supplementary Figure S1). In addition, the loading process and the biomaterials did not affect the activity of rhBMP-2. The released protein combined with its receptor on the surface of hBMSCs (Supplementary Figure S2) and led to much greater levels of calcium deposition and calcium node formation than that effected by MS/CPC scaffolds alone. Furthermore, based on the results of the histological analysis of MS/CPC/rhBMP-2 scaffolds, the blood vessels could be easily observed among the newly formed bones to form osteons. These results demonstrated outstanding bone regeneration speed with a more natural structure following the implantation of MS/CPC/rhBMP-2 scaffolds.

The nature of biomaterials, fabrication methods, and the specific cells or biomolecules largely determines the regeneration effect of bone tissue engineering scaffolds^38^,^39^. In the current study, the synthesized MS/CPC/rhBMP-2 scaffolds exhibited promising bone repair properties by providing a combination of chemicophysical and biological cues: i.e., the release of an appropriate Si ions content effectively induced neovascularization; the well-interconnected macropores encouraged the new blood vessels to grow freely inside the scaffolds to bring sufficient nutrients for regeneration; and the restrained release of rhBMP-2 from the hierarchically porous structure led to the prolonged acceleration of osteogenesis.

## Conclusions

In this study, we printed MS/CPC scaffolds containing well-interconnected macropores and ordered mesopores. The microstructure not only led to the free ingrowth of tissue but also resulted in the generation of good carriers for rhBMP-2. With the proper doping content of MS, the scaffolds exhibited good biocompatibility and effectively stimulated neovascularization. Notably, the MS/CPC/rhBMP-2 scaffolds induced the osteogenic differentiation of hBMSCs and vascularization of HUVECs *in vitro* and demonstrated abundant new vessel formation both around the scaffolds and inside the macropores as well as rapid rates of osteogenesis *in vivo* owing to the collaborative effects of the biomaterials and growth factor. In summary, the MS/CPC/rhBMP-2 scaffolds developed herein might provide a solution to the problem of tissue necrosis during bone repair through the cooperation of materials and growth factor, mediating vascularization and osteogenesis.

## Materials and Methods

### Preparation of MS powders

MS was synthesized as described^40^. Briefly, 4 g triblock copolymer P123 (Mw 5800, Sigma-Aldrich, St. Louis, MO, USA) was mixed with 150 mL HCl (1.6 M). After complete dissolution of copolymer, tetraethoxyorthosilicate (TEOS) was added drop-wise to hydrolyse for 30 min (mol ratio: TEOS/H_2_O/HCl = 1:4:0.08) and the obtained mixture was stirred for 20 h at 35 °C. Next, the suspension was transferred into a sealed reaction kettle and kept for 24 h at 110 °C without stirring. The obtained white solid was filtered and washed repeatedly with deionized water. The air-dried white powder was next calcinated at 500 °C for 6 h (heating rate 1 °C/min).

The microstructure and special surface area of MS was observed by SAXRD (Rigaku D/max 2550VB/PC, Tokyo, Japan) and via an automated surface area and pore size analyzer (BET, Novawin 4200e, Quantachrome, Beijing, China).

### Rheological properties

The effects of MS doping on the internal structure of the printing paste, which are related to suspension stability^41^, were determined by rheological property measurements using a rotational rheometer (RS600, Thermo Haake Co., Ltd., Waltham, MA, USA). After being mixed thoroughly, each sample was evenly poured into the rheometer plate platform. Mechanical spectra of the pastes were recorded at a constant deformation of 0.1% strain in the frequency range of 0.1–100 rad/s at 37 °C. Additionally, according to the calculated results of the printing pressure and the diameter of the extrude nozzle, the temperature dependent viscosity measurements were also performed (5–37 °C) at a constant shear rate of 45/s according to the calculated results of the printing pressure and the extrusion nozzle diameter.

### Fabrication and characterization of CPC and MS/CPC scaffolds

CPC (Rebone, Shanghai, China) and MS/CPC scaffolds were prepared essentially as described^7^. The CPC powder was mixed with 10 wt% MS to form MS/CPC printing powder. Printable CPC and MS/CPC pastes were prepared by mixing CPC and MS/CPC powders with the binder, 10 wt% poly(vinyl) alcohol (Mw 98,000; Sigma-Aldrich) aqueous solution. The scaffolds with the size of ϕ10 × 2 mm^3^ and 3 × 4 × 15 mm^3^ were separately printed for *in vitro* and *in vivo* experiments with the 3D Bioprinter (Qingdao Unique Co., Ltd., Qingdao, China) at room temperature. Surface topology structure and element distribution of CPC and MS/CPC scaffold were detected using SEM (Sirion 200, FEI, Hillsboro, OR, USA) and EDS (Sirion 200).

### Loading of rhBMP-2

The CPC and MS/CPC porous scaffolds were sterilized by ethylene oxide vapour in advance. RhBMP-2 (Rebone, Shanghai, China) (2 and 10 μg for cell culture or implantation, respectively) in acetic acid solution was dropped onto each scaffold and maintained in sterile conditions for 4 h to allow for total absorption. Then, the rhBMP-2-loaded scaffolds were freeze dried and stored at −20 °C for later use.

### Extract preparation

The extracts of CPC, MS/CPC, and MS/CPC/rhBMP-2 scaffold extracts were prepared according to the International Organization for Standardization method (ISO 10993-12). Briefly, the scaffolds were incubated in standard culture medium at a mass/volume rate of 100 mg/mL at 37 °C. After 24 h, the supernatant was carefully collected, filter-sterilized (0.22 mm, Millipore, Billerica, MA, USA) and stored at 4 °C (ISO10993-1) for further use^42^,^43^,^44^.

### Viability of HUVECs

HUVECs were purchased from Shanghai Institutes for Biological Science, Chinese Academy of Science (Shanghai, China) and cultured in α-minimum essential medium supplemented with 10% foetal bovine serum (FBS; Gibco BRL, Gaithersburg, MD, USA) and 1% penicillin/streptomycin (Hyclone, Logan UT, USA). To assess cell viability, 2 × 10^4^ HUVECs per well were seeded into 24-well plates and allowed to attached overnight. The culture media was then replaced with the CPC, MS/CPC, and MS/CPC/rhBMP-2 scaffold extractions. After 1, 3, and 5 days of culture, HUVEC viability was analysed via Cell Counting Kit-8 (CCK-8, Dojindo, Kumamoto, Japan). All samples were tested in triplicate and results are expressed as the means ± SD.

### *In vitro* sprouting

To measure cell sprouting, growth factor-reduced Matrigel (BD Biosciences, San Jose, CA, USA) was mixed with culture media at a ratio of 1:3 and added evenly to each well of a 96-well plate. HUVECs were separately resuspended during the extraction of CPC, MS/CPC, and MS/CPC/rhBMP-2 scaffolds and then seeded on the gel at a density of 2 × 10^4^ cells/well. HUVECs cultured in standard culture media were used as the control group. After incubation for 5 h at 37 °C in a cell culture incubator, the plate was examined by optical microscopy. At least 5 fields were digitally acquired for each substrate and the total numbers of vessels per field were counted by a blinded observer using Image pro Plus software (Media Cybernetics, Rockville, MD, USA).

### Cell culture and viability

hBMSCs were purchased from Shanghai Rochen Biotechnology Co., (Shanghai, China) and cultured in Dulbecco’s modified Eagle’s medium supplemented with 10% FBS and 1% penicillin/streptomycin at 37 °C, 5% CO_2_. To assess cell viability, 1 × 10^4^ hBMSCs per well were seeded into 24-well plates and allowed to attach overnight. The culture medium was replenished the next day with the extraction of CPC, MS/CPC, and MS/CPC/rhBMP-2 scaffolds as described in Section 2.5. The culture medium was replaced every 3 days. After 1, 3, and 5 days of culture the cell proliferation assay was performed using Cell Counting Kit-8 (CCK-8, Dojindo, Kumamoto, Japan) according to the manufacturer’s instructions. Cells cultured with standard culture medium were used as the control.

### Cell attachment

MS/CPC scaffolds (ϕ10 × 2 mm^3^) were placed in 24-well plates. Then, 1 × 10^4^ hBMSCs each were seeded onto the surface of scaffolds and cultured at 37 °C and 5% CO_2_ in a humidified incubator. After 72 h of culture, scaffolds were washed with phosphate buffer solution (PBS) and fixed with 4% paraformaldehyde (PFA). The attachment of cells onto the scaffolds was observed using SEM (Quanta 250, FEI, Hillsboro, OR, USA) after gradient elution with ethanol and thermostatic drying. Cells were also stained with FITC-Phalloidin (Sigma-Aldrich) to detect the cytoskeleton and DAPI (Sigma-Aldrich) for the nuclei. Morphological characteristics of the attached cells were observed using confocal laser scanning microscopy (Nikon, Tokyo, Japan).

### ALP activity and calcium deposition assay

To assess cell function, 5 × 10^4^ hBMSCs per well were seeded into 24-well plates and allowed to attach overnight. The culture medium was replenished the next day with the extraction of CPC, MS/CPC, and MS/CPC/rhBMP-2 scaffolds. The culture medium was replaced every 3 days. After 7 and 14 days of culture, the cells were fixed with 4% PFA, stained with an ALP kit (Beyotime, Shanghai, China), and finally observed directly and by optical microscopy. After 28 days of culture, calcium deposition was assessed by Alizarin Red S staining, for which the cells were washed with PBS, fixed with 4% PFA, and stained with 1% Alizarin Red S (Sigma-Aldrich). Afterward, the cells were washed thoroughly with PBS and observed by optical microscopy.

### *In vivo* osteointegration and vascularization

For the *in vivo* study, 54 healthy male New Zealand rabbits (6 months of age, 2.5–3.5 kg) were obtained from Shanghai SLAC Laboratory Animal Co., Ltd. (Shanghai, China) and divided into 3 experimental groups: (1) CPC, (2) MS/CPC, and (3) MS/CPC/rhBMP-2. For each group, 6 rabbits were sacrificed after implantation at 4, 8, and 12 weeks. All experiments were approved by the Department of Laboratory Animal Science, Fudan University and performed strictly in accordance with the approved guidelines.

### Animal surgical procedures

The rabbits were anaesthetized by ear intravenous injection of 1.5% pentobarbital (1 mL/kg). After the surgical site was shaved and disinfected, the radius was exposed through a longitudinal incision of the skin. Then, a defect of approximately 15 mm in height was made on the mid-shaft of the radius for scaffold implantation. To this, a 15 × 3 × 4 mm sized CPC, MS/CPC, or MS/CPC/rhBMP scaffold was implanted for bone repair. Finally, the surgical wound was carefully closed.

### Microfil perfusion

To evaluate blood vessel formation, 4-week post-implantation assessment group animals were perfused with Microfil (Flowtech, Carver, MA, USA) following euthanasia as described^45^. Briefly, after the abdominal aorta was exposed, the descending aorta was clamped and 20 mL Microfil was perfused at the appropriate speed. Radii including different scaffolds were harvested from tissue after overnight resting at 4 °C; radii were then fixed in 4% PFA for subsequent experiments.

### Sample preparation for osteogenesis assessment

At 4, 8, and 12 weeks after surgery, the animals set aside for scaffold osteogenesis ability assessment were sacrificed by air embolization. The radii including different scaffolds were harvested and trimmed to just beyond the scaffold length. All specimens were fixed with 4% PFA immediately for subsequent experiments.

### Micro-computerized tomography (μCT) analysis

All specimens were scanned for vessels and bone formation within the defects after implant retrieval using a μCT imaging system (μCT 100, SCANCO Medical AG, Bassersdorf, Switzerland). The defect sites were scanned with a resolution of 10 μm, 70 kV voltage, 200 μA current, and 300 ms exposure time. After scanning, 3D images were reconstructed using GEHC Micro-View software (GE Healthcare BioSciences, Chalfont-St. Giles, UK). The scaffolds, bone, and blood vessels were separately reconstructed and displayed by extracting all voxels with different grey value ranges.

### Histology evaluation

After fixation with 4% PFA, the radii implanted with different scaffolds were decalcified in 10% ethylenediaminetetraacetic acid, embedded in paraffin, and sectioned parallel to the long axis of the bone at a thickness of 50 μm. The sections were stained with haematoxylin/eosin (HE) and Masson trichrome and observed by light microscopy (TE2000U, Nikon, Japan). The quantity of newly formed bones was assessed using Image Pro Plus 6.0 software.

### Hard tissue slicing

After fixation with 4% PFA, the radii implanted with the different scaffolds were dehydrated with a graded series of alcohol, embedded in PMMA, and cut into 150-μm thick sections perpendicular to the scaffolds. The sections were glued onto a plastic support, polished to 50-μm thickness, stained with Van Gieson’s picro-fuchsin stain, and observed under light microscopy (TE2000U).

### Statistical analysis

The data are presented as the mean-standard deviation. Statistical analysis was performed using one-way analysis of variance (ANOVA). A value of *P* < 0.05 was considered statistically significant.

## Additional Information

**How to cite this article**: Li, C. *et al*. RhBMP-2 loaded 3D-printed mesoporous silica/calcium phosphate cement porous scaffolds with enhanced vascularization and osteogenesis properties. *Sci. Rep.* **7**, 41331; doi: 10.1038/srep41331 (2017).

**Publisher's note:** Springer Nature remains neutral with regard to jurisdictional claims in published maps and institutional affiliations.

## Supplementary Material

Supplementary Information
