# Supplementary material for: RhBMP-2 loaded 3D-printed mesoporous silica/calcium phosphate cement porous scaffolds with enhanced vascularization and osteogenesis properties
Source: Sci Rep. 2017 Jan 27;7:41331. doi: 10.1038/srep41331 (PMC5269721; doi:10.1038/srep41331)
Supplement: Supplementary Information [file srep41331-s1.doc]

**Support Information**

**RhBMP-2 loaded 3D-printed mesoporous silica/calcium phosphate cement porous scaffolds with enhanced vascularization and osteogenesis properties**

Cuidi Li,^1,2,†^ Chuan Jiang,^3,†^ Yuan Deng,^1,2^ Tao Li,^2^ Ning Li,^1^ Mingzheng Peng^2^ & Jinwu Wang^1,2,*^

^1^School of Biomedical Engineering, Shanghai Jiao Tong University, Shanghai, China.

^2^Shanghai Key Laboratory of Orthopaedic Implants, Shanghai Ninth People's Hospital Affiliated Shanghai Jiao Tong University School of Medicine, Shanghai, China.

^3^Department of Orthopaedics, Sun Yat-sen Memorial Hospital, Sun Yat-sen University, 107 West Yanjiang Road, Guangzhou, China.

^†^These authors contributed equally to this work.

*Correspondence and requests for materials should be addressed to J. W. (jinwu_wang@163.com)

**Figure S1: Release kinetics of rhBMP-2 from various scaffolds**


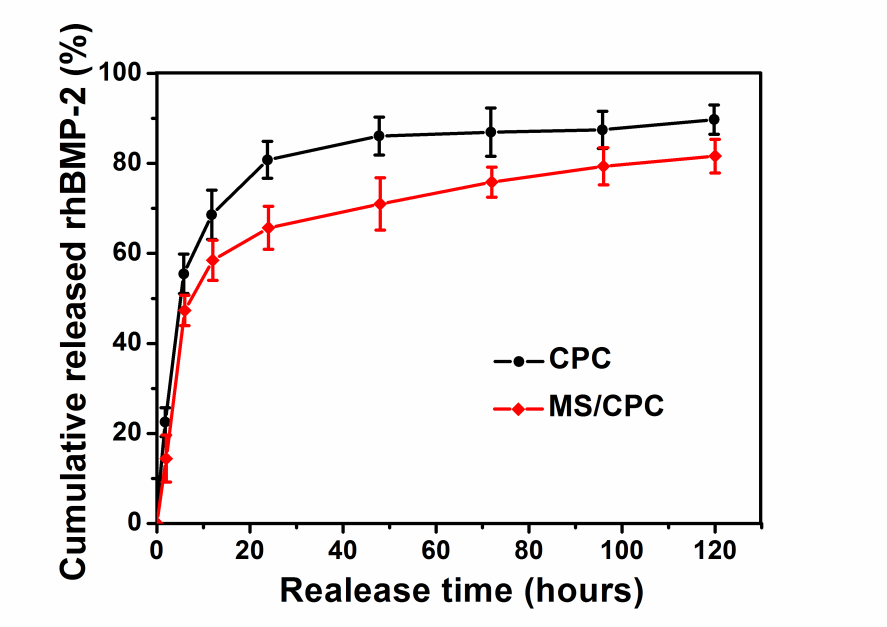


Cumulative release of rhBMP-2 from CPC and MS/CPC scaffolds in SBF.

**Figure S2: Binding capacity of rhBMP-2 to cellular receptors**


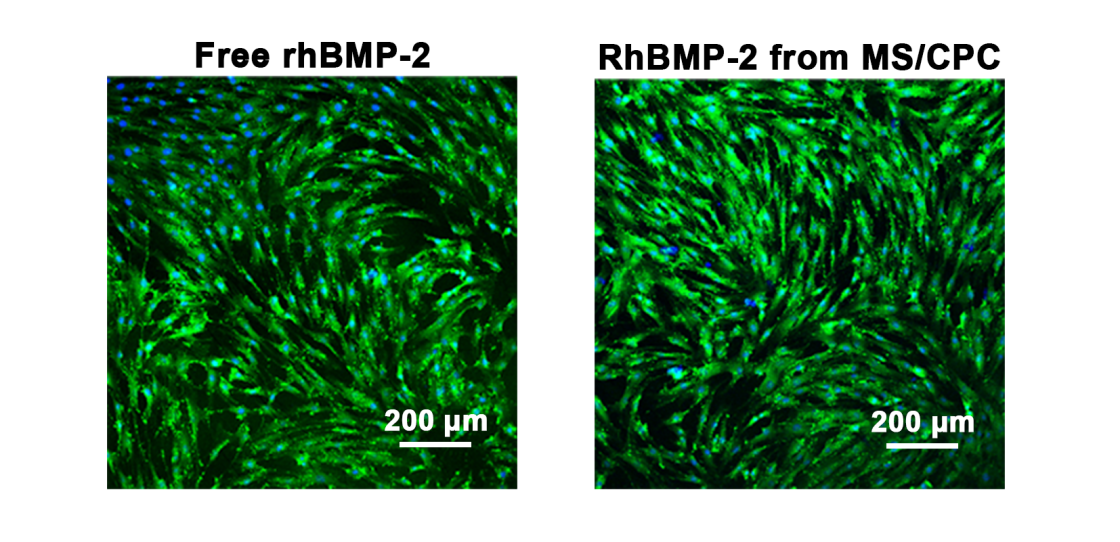


The binding capability of free rhBMP-2 and released rhBMP-2 stained with anti-BMP-2-FITC on the hBMSCs with nuclei stained with DAPI after 10 h of culture.
